# The Monocyte‐to‐Lymphocyte Ratio Was Associated With Intraplaque Neovascularization of the Carotid Artery on AngioPLUS

**DOI:** 10.1002/brb3.70058

**Published:** 2024-09-30

**Authors:** Mingfeng Zhai, Xiao Sun, Jian Wang, Jimei Xu, Fuqin Bian, Menglin Wu, Yafei Yang, Hongwei Chen, Jinghong Lu

**Affiliations:** ^1^ Department of Neurology The Affiliated Fuyang People's Hospital of Anhui Medical University Fuyang China; ^2^ Department of Neurology Hefei Hospital Affiliated to Anhui Medical University Hefei China; ^3^ Department of Ultrasound Hefei Hospital Affiliated to Anhui Medical University Hefei China; ^4^ Fuyang Medical College Fuyang Normal University Fuyang Anhui China

**Keywords:** angio planewave ultrasensitive imaging, carotid plaque, intraplaque neovascularization, lymphocytes, monocyte–lymphocyte ratio, monocytes

## Abstract

**Background:**

The monocyte–lymphocyte ratio (MLR) is a hematological test parameter that reflects the status of both monocytes and lymphocytes as inflammatory cells. This study aims to investigate the relationship between MLR and carotid intraplaque neovascularization (IPN) in patients with asymptomatic carotid stenosis.

**Methods:**

We performed the Angio Planewave Ultrasensitive (AngioPLUS) screening for patients with carotid plaques. The carotid plaque stability was evaluated by semiquantitative visual grading of carotid IPN. Binary logistic regression models were performed to determine the associations between different clinical and laboratory indicators and the presence of high IPN.

**Results:**

A total of 160 patients were eventually enrolled with 99 in the low IPN group (Scores 0–1) and 61 in the high IPN group (Score 2). Univariate logistic regression showed that age, monocytes, lymphocytes, glycated hemoglobin (HbA1c), fibrinogen, d‐dimmer, and MLR were significantly associated with the presence of high IPN (all *p* < 0.05). Multivariate logistic regression models showed that MLR was significantly associated with the presence of high IPN after adjusting for other covariates. An MLR value of 32.9 was the optimal cutoff value to differentiate high and low IPN. High MLR was also significantly correlated with the presence of high IPN (odds ratio [OR] = 4.08, 95% confidence interval [CI]: 1.69–9.88, *p* = 0.002) when included in the above multivariate logistic regression model.

**Conclusion:**

Elevated MLR is closely associated with the presence of high IPN and may serve as a surrogate biomarker for carotid IPN.

## Graphical abstract

The monocyte–lymphocyte ratio (MLR) is a hematological test parameter that reflects the status of both monocytes and lymphocytes as inflammatory cells. We found that elevated MLR is closely associated with the presence of high carotid intraplaque neovascularization (IPN) and may serve as a surrogate biomarker for carotid IPN.

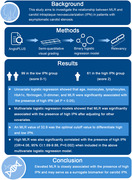

## 1 Introduction

Carotid arteries are the “windows” to the human arteries, and the role of the pathological characteristics and vulnerability of carotid plaques in the pathogenesis of ischemic stroke is highly valued. However, it is not conclusive whether all carotid plaques require statin intervention. Compared to stable plaques, vulnerable plaques are at a relatively higher risk of causing ischemic stroke. Intraplaque neovascularization (IPN) is naturally of particular interest as a feature of carotid plaque vulnerability (Ding et al. 2008). Carotid IPN can increase intraplaque hemorrhage, which in turn promotes carotid plaque rupture and dislodgement, and is closely associated with the development of ischemic strokes (Koole et al. 2012). The methods used to detect IPN in plaques include contrast‐enhanced ultrasonography (CEUS), superb microvascular imaging (SMI), and angio planewave ultrasensitive (AngioPLUS) imaging (Hamada et al. 2016; Zamani et al. 2019; Chen et al. 2022). However, CEUS is not widely available in clinical practice due to its high price, advanced technical requirements, and the presence of contrast allergies (Yang and Wang 2020). AngioPLUS technology is a new microvascular Doppler ultrasound technique with higher resolution, which allows for the detection of even smaller vessels and blood flow signals with lower flow rates (Liu et al. 2019). Further semiquantitative grading of carotid IPN helps to assess the vulnerability of carotid plaque.

Identifying associated risk factors or biomarkers that influence the formation and development of carotid IPN is particularly important for guiding further interventions.

Inflammation plays an important role in the development and instability of atherosclerotic plaques (McCabe et al. 2021). The monocyte–lymphocyte ratio (MLR) is a hematological test parameter that can be obtained simply from routine blood tests and reflects the status of both monocytes and lymphocytes as inflammatory cells in the body. Monocytes can differentiate into macrophages and dendritic cells, which are the main cells involved in innate immunity, whereas lymphocytes play an important role primarily in specific immunity (Wang and Xia 2013; Matveeva and Grigoriev 2016). Thus, these cells mediate two different immune or inflammatory pathways and provide more comprehensive information than individual leukocyte parameters. Previous studies have found that the MLR is also an independent risk factor for carotid artery diseases and poststroke depression (Xiang et al. 2018; Ding et al. 2021). For example, the higher MLR value is related to the degree of carotid artery stenosis, and it is an independent risk factor for carotid artery stenosis (Zuo et al. 2019). However, the role of MLR in carotid IPN has not yet been fully elucidated. We hypothesized that investigation of the relationship between MLR and IPN may facilitate an understanding of the inflammatory mechanism in the pathogenesis of carotid IPN. This study aims to evaluate the role of readily available inflammatory biomarkers monocyte, lymphocyte, and the derived MLR in the formation of carotid IPN.

## 2 Methods

### 2.1 Study Design and Patients

We enrolled consecutive asymptomatic patients who underwent the AngioPLUS screening for carotid plaques at our hospital from October 2020 to April 2022. All patients or their guardians provided their written informed consent to participate in this study. We included patients aged > 18 years with carotid plaque formation (plaque thickness ≥ 1.5 mm) on 2D ultrasound images and AngioPLUS examination for carotid plaques. We excluded patients who had undergone carotid stenting or carotid endarterectomy, had a history of malignancy, or failed to complete the AngioPLUS examination due to various factors (such as obesity, abnormal carotid artery course, severe dementia, and psychiatric factors).

### 2.2 Clinical Data

We collected demographic information, including age and sex; medical history records, including hypertension, diabetes, dyslipidemia, and current smoking or drinking; body mass index and blood pressure (systolic and diastolic) at admission. Hypertension was determined by prior use of antihypertensive drugs, systolic blood pressure (SBP) ≥ 140 mmHg or diastolic blood pressure (DBP) ≥ 90 mmHg. Diabetes was determined by prior use of antidiabetic drugs, fasting blood glucose (FBG) ≥ 7.0 mmol/L, or 2‐h postprandial blood glucose ≥ 11.1 mmol/L.

### 2.3 Laboratory Indicators

Fasting blood samples were collected on the morning of the second day after admission following an overnight fast. Blood tests were determined using an automated blood cell analyzer (XN‐10 Sysmex Company, Japan). Fibrinogen and d‐dimer levels in plasma were measured using an automatic coagulation analyzer (Stago STAR Max, France). FBG, triglycerides (TG), total cholesterol (TC), and other biochemical parameters were assayed using an automatic biochemical analyzer (HITACHI Automatic Analyzer 7600‐020, Japan). The MLR was calculated using the following formula: MLR = monocyte/lymphocyte × 10^−2^.

### 2.4 Evaluation of Carotid IPN

We first perform a routine 2D ultrasound examination. The subject is told to lie supine with full exposure of the neck. The internal–medial thickness is first measured along the course of the carotid artery; then we observe the presence of plaque in the common carotid artery and its bifurcations, the internal and external carotid arteries, and make detailed notes on the location, size (measuring the maximum thickness diameter and maximum length diameter of the plaque), number, and echogenicity of the plaque. We then used the French SuperSonic Imagine AixPlorer Imaging Ultrasound Diagnostic Instrument (Sonic Red Series, built‐in AP ultrasound imaging technology, equipped with SL10‐2 probe) to observe and evaluate the IPN of the target plaque. The number, location and shape of the carotid IPNs (e.g., dots and short strips), and the flow spectral characteristics are recorded in detail. The above examinations were assessed double‐blind by two trained sonologists. If any disagreement arises, a third senior sonologist is consulted to resolve the issue. In addition, the sonologists were blinded to the laboratory data of enrolled patients.

### 2.5 Statistical Analysis

Statistical analysis was performed using SPSS 24.0 (SPSS Inc., Chicago, IL, USA) software. Baseline clinical data and laboratory indicators were presented as mean ± standard deviation or median (inter‐quartile range) for continuous variables and percentage for categorical variables. Differences in continuous variables between groups were assessed by Student's *t*‐test or Mann–Whitney *U*‐test. Differences in categorical variables between groups were assessed by the *χ* ^2^ test or Fisher's exact test. Univariate and multivariate logistic regressions were performed to determine the associations between different variables and IPN scores. The odds ratio (OR) and 95% confidence interval (CI) were subsequently calculated. Statistical significance was taken as *p* < 0.05. All plots were drawn using GraphPad Prism software (version 8.0).

## 3 Results

### 3.1 Baseline Characteristics

A total of 160 patients were eventually enrolled, including 95 males and 65 females, with a median age of 68 years. In terms of risk factors for vascular disease, there were 129 patients (80.6%) with hypertension, 41 patients (25.6%) with diabetes, and 30 patients (18.6%) with dyslipidemia (Table 1). Among these patients, 82 patients had bilateral carotid plaques and 78 patients had unilateral carotid plaques, whereas 99 were categorized as the low IPN group (Scores 0–1) and 61 as the high IPN group (Score 2).

**TABLE 1:** Clinical and laboratory indicators between high and low intraplaque neovascularization (IPN) groups.

Variables	All patients (*n* = 160)	Low IPN (*n* = 61)	High IPN (*n* = 99)	Unadjusted OR (95% CI)	*p* value
Age (years)	68 (13)	67 (14)	70 (12)	1.03 (1.0–1.06)	0.011
Male, *n* (%)	95 (59.4)	33 (54.1)	62 (62.6)	1.42 (0.74–2.72)	0.286
Hypertension, *n* (%)	129 (80.6)	46 (75.4)	83 (83.8)	1.69 (0.77–3.73)	0.190
Diabetes mellitus, *n* (%)	41 (25.6)	12 (19.7)	29 (29.3)	1.69 (0.79–3.64)	0.176
Dyslipidemia, *n* (%)	30 (18.6)	9 (14.8)	21 (21.2)	1.56 (0.66–3.66)	0.309
Current drinking, *n* (%)	37 (23.1)	11 (18.0)	26 (26.3)	1.62 (0.73–3.57)	0.230
Current smoking, *n* (%)	31 (19.4)	9 (14.8)	22 (22.2)	1.65 (0.70–3.87)	0.246
BMI (kg/m^2^)	24.15 ± 2.60	24.01 ± 2.60	24.23 ± 2.61	1.03 (0.91–1.17)	0.597
Neutrophils (%)	60.2 (13.2)	59.4 (13.4)	61.4 (13.1)	1.20 (0.99–1.06)	0.160
Lymphocyte (%)	28.73 ± 8.46	30.97 ± 8.09	27.35 ± 8.43	0.95 (0.91–0.99)	0.008
Monocyte (%)	7.70 ± 2.64	7.00 ± 2.91	8.13 ± 2.37	1.19 (1.04–1.35)	0.008
Platelet (×10^9/L)	164 (60)	164 (51)	167 (71)	1.00 (0.99–1.01)	0.848
FBG (mmol/L)	5.2 (1.6)	5.1 (1.2)	5.4 (1.7)	1.20 (0.99–1.44)	0.240
HbA1c (%)	5.9 (1.3)	5.5 (1.0)	6.1 (1.3)	1.72 (1.20–2.46)	< 0.001
TC (mmol/L)	4.28 ± 0.97	4.32 ± 1.05	4.25 ± 0.92	0.93 (0.67–1.30)	0.685
TG (mmol/L)	1.43 (1.10)	1.36 (1.30)	1.46 (1.08)	0.95 (0.77–1.17)	0.484
HDL (mmol/L)	1.10 (0.34)	1.12 (0.33)	1.05 (0.34)	0.69 (0.22–2.20)	0.415
LDL (mmol/L)	2.45 ± 0.77	2.47 ± 0.85	2.44 ± 0.73	0.95 (0.63–1.44)	0.813
CRP (mg/L)	3.6 (7.5)	3.6 (5.1)	3.6 (8.5)	1.04 (0.98–1.11)	0.413
HCY (µmol/L)	13.6 (6.5)	12.6 (5.2)	14.3 (6.6)	0.99 (0.96–1.02)	0.064
Uric acid (µmol/L)	335 (106)	328 (138)	340 (102)	1.00 (0.99–1.01)	0.961
Fibrinogen	3.11 (0.71)	2.93 (0.65)	3.17 (0.87)	3.31 (1.73–6.34)	< 0.001
d‐Dimmer (µg/mL)	0.36 (0.32)	0.34 (0.21)	0.44 (0.36)	6.40 (1.52–26.90)	0.004
MLR (10^−2^)	26.09 (16.75)	23.12 (13.59)	29.64 (16.61)	5.09 (2.19–11.82)	< 0.001

*Note*: Numbers are given as mean ± standard deviation (SD), median (inter‐quartile range) for continuous variables, and percentage for categorical variables.

Abbreviations: BMI, body mass index; CI, confidence interval; CRP, C‐reactive protein; FBG, fasting blood glucose; HbA1c, glycated hemoglobin; HCY, homocysteine; LDL, low‐density lipoprotein; MLR, monocyte–lymphocyte ratio; OR, odds ratio; TC, total cholesterol; TG, triglycerides.

### 3.2 Comparison of Clinical and Laboratory Indicators Between High and Low IPN Groups

Age (*p* = 0.011), monocytes (*p* = 0.008), glycated hemoglobin (HbA1c) (*p* < 0.001), fibrinogen (*p* < 0.001), d‐dimmer (*p* = 0.004), and MLR levels (*p* < 0.001) were higher in the high IPN group compared to those in the low IPN group, whereas the lymphocyte count was significantly lower than that in the low IPN group (*p* = 0.008). The difference in other valuables was not statistically significant between two groups (*p* > 0.05).

### 3.3 The MLR Was Associated With The Presence of High IPN

We used binary logistic regression models to analyze potential factors associated with the presence of high IPN. Univariate logistic regression showed that age, monocytes, lymphocytes, HbA1c, fibrinogen, d‐dimmer, and MLR were significantly associated with the presence of high IPN (all *p* < 0.05) (Table 1). Multivariate logistic regression models showed that monocytes, lymphocytes, HbA1c, fibrinogen, and MLR were significantly associated with the presence of high IPN (all *p* < 0.05) (Table 2).

**TABLE 2:** Multivariate logistic regression models of associated factors for high intraplaque neovascularization (IPN).

Models	Adjusted OR (95% CI)	*p* value
**Model 1 (with monocyte)**		
Monocyte	1.21 (1.05–1.40)	0.008
HbA1c	1.66 (1.14–2.42)	0.008
Fibrinogen	2.88 (1.45–5.69)	0.002
**Model 2 (with lymphocyte)**		
Lymphocyte	0.952 (0.91–0.99)	0.029
HbA1c	1.60 (1.12–2.23)	0.009
Fibrinogen	2.76 (1.42–5.36)	0.003
**Model 3 (with monocyte and lymphocyte)**		
Lymphocyte	0.94 (0.90–0.99)	0.011
Monocyte	1.25 (1.08–1.46)	0.003
HbA1c	1.68 (1.17–2.42)	0.005
Fibrinogen	2.73 (1.36–5.49)	0.005
**Model 3 (with MLR)**		
HBA1C	1.63 (1.14–2.34)	0.008
Fibrinogen	2.57 (1.29–5.17)	0.007
MLR	1.06 (1.03–1.10)	0.001

*Note*: Potential risk factors with a *p* value of < 0.1 in the univariate analysis or clinically relevant factors were included in the multivariate logistic regression models with a forward stepwise procedure. Model 1: Age + monocyte + HbA1c + HCY + d‐dimmer + fibrinogen; Model 2: age + lymphocyte + HbA1c + HCY + d‐dimmer + fibrinogen; Model 3: age + lymphocyte + monocyte + HbA1c + HCY + d‐dimmer + fibrinogen; Model 4: age + HbA1c + HCY + d‐dimmer + fibrinogen + MLR.

Abbreviations: 95% CI, 95% confidence interval; HbA1c, glycated hemoglobin; MLR, monocyte–lymphocyte ratio; OR, odds ratio.

### 3.4 Clinical and Laboratory Indicators Compared Between High and Low MLR Groups and the Relationship Between High MLR and High IPN

**FIGURE 1:**
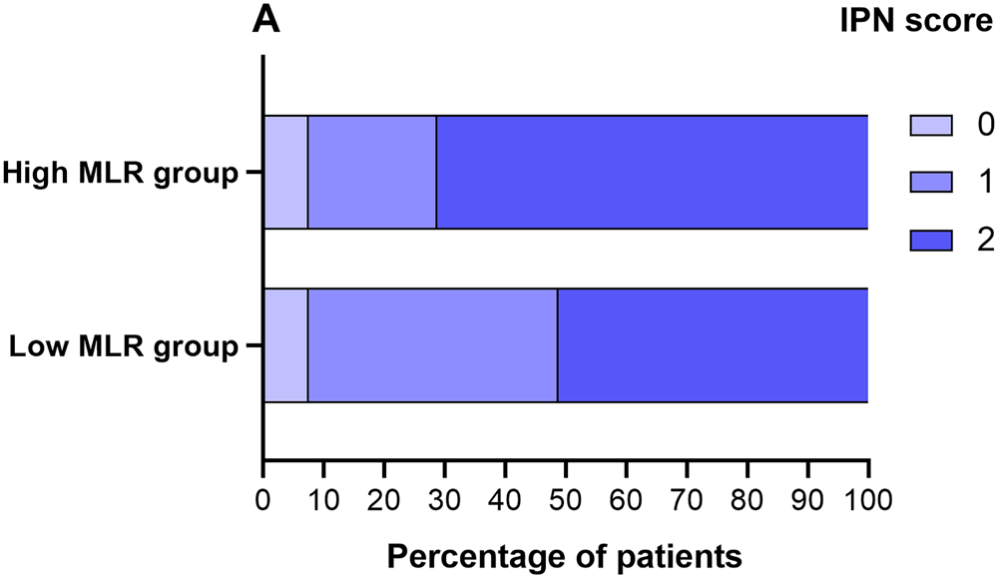
The IPN score distribution between the high MLR group and low MLR group. IPN, intraplaque neovascularization; MLR, monocyte–lymphocyte ratio.

Using the MLR cutoff value of 32.9, we divided our patients into two groups: a low MLR group (MLR < 32.9) and a high MLR group (MLR ≥ 32.9). The characteristics of these two groups are summarized in Table 3 and Figure 1. Compared to the low MLR group, patients in the high MLR group had a higher proportion of male (*p* = 0.048) and neutrophils (*p* < 0.001), monocyte (*p* < 0.001), HbA1c (*p* = 0.042), C‐reactive protein (CRP) (*p* = 0.010), homocysteine (HCY) (*p* = 0.004), fibrinogen (*p* = 0.012), and d‐dimer levels (*p* = 0.004); however, the lymphocyte ratio (*p* < 0.001) was lower than that in the high MLR group (Table 3). In addition, high MLR was also significantly associated with the presence of high IPN (OR = 5.09, 95% CI: 2.19–11.82, *p* < 0.001) when the variable MLR grouping was included in univariate logistic regression. High MLR was also significantly correlated with the presence of high IPN (OR = 4.08, 95% CI: 1.69–9.88, *p* = 0.002) when the variable MLR grouping was included in the above multivariate logistic regression model.

**TABLE 3:** Clinical and laboratory indicators between high and low monocyte–lymphocyte ratio (MLR) groups.

Variables	All patients (*n* = 160)	Low MLR (*n* = 109)	High MLR (*n* = 51)	*p* value
Age (years)	68 (13)	68 (14)	70 (11)	0.101
Male, *n* (%)	95 (59.4)	59 (54.1)	36 (70.6)	0.048
Hypertension, *n* (%)	129 (80.6)	89 (81.7)	40 (78.4)	0.631
Diabetes mellitus, *n* (%)	41 (25.6)	29 (26.6)	12 (23.5)	0.678
Dyslipidemia, *n* (%)	30 (18.8)	22 (20.2)	8 (15.7)	0.497
Current drinking, *n* (%)	31 (19.4)	18 (16.5)	13 (25.5)	0.181
Current smoking, *n* (%)	37 (23.1)	24 (22.0)	13 (25.5)	0.627
BMI (kg/m^2^)	24.15 ± 2.60	24.12 ± 2.52	24.19 ± 2.79	0.884
Neutrophils (%)	60.2 (13.3)	57.4 (13.3)	64.4 (10.6)	< 0.001
Lymphocyte (%)	28.73 ± 8.46	31.67 ± 7.75	22.45 ± 6.24	< 0.001
Monocyte (%)	7.70 ± 2.64	6.76 ± 2.22	9.71 ± 2.32	< 0.001
Platelet (×10^9^/L)	164 (40)	165 (55)	163 (78)	0.969
FBG (mmol/L)	5.23 (1.55)	5.23 (1.27)	5.36 (1.79)	0.746
HbA1c (%)	5.9 (1.3)	5.8 (1.3)	6.2 (1.0)	0.042
TC (mmol/L)	4.28 ± 0.97	4.36 ± 1.02	4.10 ± 0.83	0.114
TG (mmol/L)	1.42 (1.10)	1.45 (1.17)	1.30 (0.90)	0.349
HDL (mmol/L)	2.45 ± 0.77	1.11 (0.41)	1.08 (0.25)	0.873
LDL (mmol/L)	2.45 ± 0.77	2.53 ± 0.83	2.29 ± 0.61	0.073
CRP (mg/L)	3.6 (7.5)	3.1 (5.5)	5.1 (8.4)	0.010
HCY (µmol/L)	13.6 (6.5)	13.0 (5.9)	15.2 (7.0)	0.004
Uric acid (µmol/L)	335 (106)	322 (131)	349 (85)	0.063
Fibrinogen	3.11 (0.71)	3.06 (0.67)	3.18 (1.10)	0.012
d‐Dimmer (µg/mL)	0.36 (0.32)	0.35 (0.29)	0.45 (0.45)	0.004

Abbreviations: BMI, body mass index; CRP, C‐reactive protein; FBG, fasting blood glucose; HbA1c, glycated hemoglobin; HCY, homocysteine; LDL, low‐density lipoprotein; TC, total cholesterol; TG, triglycerides.

## 4 Discussion

The present study found that MLR was closely associated with carotid IPN scores on AngioPLUS. In addition, HbA1c and fibrinogen were also associated with carotid IPN scores. Therefore, elevated MLR may serve as a surrogate indicator for carotid IPN.

The development of carotid atherosclerosis is a process of chronic inflammation and lipid accumulation. Many studies have demonstrated that the development of carotid atherosclerosis is often accompanied by an infiltration of inflammatory cells, such as macrophages and T cells (Chan and Ramji 2020; Song et al. 2020). Disturbances in the inflammatory response are the driving factor in carotid atherosclerosis formation and plaque instability (Ortega‐Paz, Capodanno, and Angiolillo 2021). Inhibition of inflammatory cytokines significantly reduces the risk of carotid plaque development (Shah 2019; Tuñón et al. 2018). Vulnerable plaques are plaques that have a propensity to rupture, are prone to thrombosis, and/or may rapidly develop into responsible lesions on the basis of carotid atherosclerosis and are closely associated with ischemic strokes (Gonçalves et al. 2015). The carotid IPN is a small vessel surrounded by relatively simple endothelial cells and lacks the corresponding connective tissue and basal cell membrane support. As a result, its walls are brittle and are prone to rupture and bleed under the repeated impact of blood flow, resulting in thrombosis and even cerebral infarction. IPN can promote the development of atherosclerotic lesions and even induce intraplaque hemorrhage and plaque rupture, and is an important factor contributing to plaque vulnerability (Boswell‐Patterson et al. 2021).

Monocytes and lymphocytes are key cells in the leukocyte subpopulation involved in the inflammatory process. MLR is a marker of inflammation reflecting monocyte and lymphocyte counts, which reflects the status of systemic inflammation and represents the degree of activation of the immune response in the body (Wolf and Ley 2019; Marcuzzi et al., 2021). A decrease in absolute lymphocyte counts and an increase in absolute d monocyte counts lead to an increase in MLR, resulting in an imbalance between innate and adaptive immunity, which may be the main cause of arterial plaque formation (Zeynalova et al. 2021). Ma et al. found that leukocyte‐derived ratios, including neutrophil‐to‐lymphocyte ratio (NLR), dNLR, MLR, platelet‐to‐lymphocyte ratio (PLR), white blood cell count–to–mean platelet volume ratio (WMR), systemic immune–inflammation index (SII), and systemic inflammatory response index (SIRI), were significantly associated with carotid plaque formation, especially MLR. In particular, patients with coronary artery disease are more likely to develop carotid plaque when the MLR is ≥ 0.35 (Ma et al. 2022). The present study found that MLR was also significantly associated with IPN, further confirming the relationship between the inflammatory response and carotid plaque instability. Monocytes can accumulate, adhere, and differentiate into inflammatory dendritic cells, macrophages, and foam cells under chemotaxis and then activate the secretion of pro‐inflammatory cytokines, matrix metalloproteinases, and reactive oxidation substances, which play a key role in the formation and development of atherosclerotic plaques (Kounis et al. 2021). Lymphocytes can regulate the phenotype of monocytes, induce the expression of tissue inhibitors of metalloproteinases, and inhibit the growth and rupture of atherosclerotic plaques, which are protective factors against atherosclerosis (Fiedorczyk et al. 2006). MLR combines an increase in the risk factor of monocytes with a decrease in the protective factor of lymphocytes, which has a dual risk effect on the formation of carotid plaque (Wolf and Ley 2019).

In addition, we found that HbA1c and fibrinogen were also associated with carotid IPN. Previous studies have also suggested that carotid IPN frequently occurred in asymptomatic patients with intermediate carotid stenosis and was more prevalent in those with diabetes (Magnoni et al. 2019). The increased production of reactive oxygen species and the direct toxic effects of hyperglycemia in the diabetic state may lead to reduced release of stromal cell–derived factor 1 (SDF1) and vascular endothelial growth factor (VEGF), suppressed activation of their downstream signaling molecules, and reduced nitric oxide bioavailability, resulting in vasodilatory dysfunction, accelerating endothelial cell apoptosis, and suppressing proliferation and migration (Catrina et al. 2004; Jarajapu 2020).

Fibrinogen is one of the key factors in the coagulation reaction. As the coagulation reaction proceeds, fibrinogen is converted into fibrin, and fibrin degradation products are deposited in the vascular wall, which, on the one hand, directly destroys vascular endothelial cells, promotes smooth muscle cell proliferation and migration, and facilitates lipid infiltration and accumulation; on the other hand, it regulates the adhesion and migration of inflammatory cells, stimulates endothelial cells and inflammatory cells to secrete a variety of inflammatory factors and other active substances, and induces inflammatory and immune responses. These processes constantly aggravate the process of atherosclerosis, promote the formation, progression, and rupture of plaques, increase their vulnerability, and participate in the formation of atherosclerotic plaques (Jaakkola et al. 2018; Cao et al. 2013). The vulnerability of plaques depends mainly on the degree of intraplaque vascularization, and the present study also found that fibrinogen was also associated with carotid IPN (Hjelmgren et al. 2018).

The present study is a single‐center cross‐sectional study in which we demonstrated that MLR can be used to predict carotid IPN by constructing multiple logistic regression models with convincing results. However, several limitations should be considered when interpreting the results of this study. First, this was a single‐center observational study with limited sample size and geographical limitations, which may have some selection bias. It needs to be verified in future studies with a larger, preferably multicenter sample. This cross‐sectional study can only demonstrate that MLR is associated with carotid IPN and cannot draw causal conclusions, and we will demonstrate this relationship in future prospective studies. Third, the mechanisms of how MLR participates in carotid IPN formation are not yet fully understood. Therefore, future basic research on this issue is required. However, to our knowledge, the current study is relatively novel in that it evaluates, for the first time, the relationship between MLR and the occurrence of carotid IPN. From a clinical point of view, we may consider MLR as a simple biomarker of carotid IPN.

## 5 Conclusion

In conclusion, this study demonstrates the close relationship between the readily available inflammatory biomarkers monocytes, lymphocytes, and derived MLR and carotid IPN formation on AngioPLUS. Elevated MLR may serve as a surrogate biomarker for carotid IPN.

## Author Contributions

**Jinghong Lu** and **Mingfeng Zhai**: conceptualization, funding acquisition, supervision, writing–original draft, writing–review and editing. **Jian Wang**: conceptualization, data curation, investigation, methodology and software. **Jimei Xu**, **Fuqin Bian**, **Menglin Wu**, and **Yafei Yang**: data curation, formal analysis, investigation and validation. **Xiao Sun** and **Hongwei Chen**: formal analysis, investigation, methodology and validation. All authors approved the protocol.

## Ethics Statement

The study was performed according to the Declaration of Helsinki guidelines and was approved by the Institutional Review Board of the Affiliated Hefei Hospital of Anhui Medical University (approval number: [2022] 077).

## Consent

All patients gave informed consent prior to their inclusion in the study. Informed consent was obtained directly from the patient or from a family member. All patients or their close relatives were informed of the purpose of the study.

## Conflicts of Interest

The authors declare no conflicts of interest.

### Peer Review

The peer review history for this article is available at https://publons.com/publon/10.1002/brb3.70058.

## Data Availability

All data generated for this study are included in the article. The datasets generated during the current study are available from the corresponding author on reasonable request.
